# “I am anxious and desperate”: psychological experiences of women with infertility in The Greater Accra Region, Ghana

**DOI:** 10.1186/s40738-017-0033-1

**Published:** 2017-03-16

**Authors:** Ernestina S. Donkor, Florence Naab, Deborah Y. Kussiwaah

**Affiliations:** 1 Department of Maternal and Child Health, School of Nursing, College of Health Sciences, University of Ghana, Accra, Legon Ghana; 2 Ga South Municipal District Hospital, Weija, Accra Ghana

**Keywords:** Experience, Infertility, Psychological, Women

## Abstract

**Background:**

Research has shown that infertility affects millions of couples worldwide. Infertility is considered one of the most difficult life experiences and can result in psychological consequences for couples, especially for women. The purpose of this study was to explore the psychological experiences of women with infertility.

**Methods:**

A qualitative exploratory descriptive approach was used to conduct in-depth interviews. The psychological component of the bio-psychosocial model was used as a guiding framework to understand the experiences of women with infertility. Fourteen women were selected based on the purpose of the study and provided their informed consent, and were interviewed between November 2015 and January 2016. All the women were recruited from the Greater Accra Region of Ghana. Each interview lasted for 30 to 40 min. All interviews were audio taped, transcribed verbatim and analyzed using thematic content analysis.

**Results:**

The findings from the study showed that women with fertility problems experienced many emotional difficulties such as loneliness, anxiety, depression, lack of concentration, worrying, and reduced sexual satisfaction.

**Conclusions:**

Women in this study encountered psychological challenges and experienced emotional distress. Such women would benefit from psychological support such as counseling to help alleviate their psychological problems. These findings have implications for the care of women with infertility in Ghana.

## Background

In Africa, motherhood is used as a measure of a woman’s reputation in society whiles it is also considered as a source of power, pride and an important aspect of life. This therefore means that African women with fertility problems experience many psychological stressors wherever they find themselves [1, 2].

In Ghana and in some parts of Africa, childbearing is seen as one of the major transitional periods in adult life for both men and women. Infertility is also seen as a major life problem in most homes and sometimes leads to psychological stressors such as marital instability, sexual dysfunction with decreased quality of life, less stable marital relationships and lower sexual satisfaction as compared to other fertile females [3, 4]. There is also some evidence that infertility is often associated with a great number of negative emotions and experiences like stigma, anxiety, and depression that go a long way to affect several aspects of lives including religious faith, self esteem, occupation, relationship with partners and that of family and friends [5–7].

In addition to the above mentioned stressful experiences, research has shown that intimacy in the lives of couples may change with a diagnosis of infertility and this makes a difference in the way couples manage their relationship, particularly their sexual life. It is believed that regardless of the causes of infertility, after the diagnosis, there may be an alteration in the mode of communication among couples leading to a decrease in libido and changes in mood [8, 9].

In view of the numerous emotional traumas experienced by women with fertility problems, the psychological component of the bio-psychosocial framework by Engel was adapted as an organizing framework to understand the psychological experiences of women with fertility problems. The psychological component of the bio-psychosocial model addresses the psychological problems as human behavior and mental processes. The theory views the individual in relation to his or her cognition, feelings or emotions. These are observable psychological attributes among individuals with any illness.

The theory further posited that although disease is mainly viewed from a medical perspective, its consequences or outcomes go beyond medicine as it has a broader impact on all aspects of life, because of anxiety, sadness, guilt, shame, hopelessness, emptiness, loss, loneliness, and depression. This however means that every sick person experiences some form of psychological trauma. Few studies on infertility have utilized this framework. Moreover, a few quantitative studies on the psychological experiences of women with fertility problems conducted in Karachi-Pakistan and Ghana revealed that women with infertility scored significantly high on depression and anxiety questions as compared to fertile women, leading to blame and other serious psychological problems [6, 10–12]. Although Engel [13] affirmed in the bio-psychosocial model that health should be examined and managed based on biological, psychological and social factors, it is not so when it comes to issues of infertility. This study therefore explored the psychological experiences of women with infertility using the psychological component of the bio-psychosocial model as an organizing framework.

## Methods

A qualitative exploratory descriptive approach was used to explore the psychological experiences of women with infertility receiving treatment in a tertiary hospital in the Greater Accra region of Ghana. Ethical clearance was sought from the Institutional Review Board of Noguchi Memorial Institute for Medical Research (IRB-NMIMR) in the University of Ghana.

With the help of the matron in charge, the purpose of the study, inclusion criteria, and the contents of the consent form were explained to volunteers. Each of the women was given a participant information sheet that had details about the study. At the end of the meeting, 18 volunteers who were seeking treatment for infertility were recruited but the data saturated (that is, where no new information was generated) at the 14th participant. Thus, data collection ended at the 14^th^ interview. On the day of the interview, each interested volunteer signed a consent form. The day, time and venue for the interview were scheduled at their convenience.

In-depth interviews were conducted between November 2015 and January 2016. Most interviews (12) were conducted in the hospital facility, with just two conducted in the homes of the women. Prior to the interview, the efficiency of the interview guide was tested on four women with infertility but recruited from a different hospital. Interviews were conducted in Ga, Akan and English and contained questions on the psychological experiences of infertility. The interviews were carried out in designated places of each participant’s choice where privacy was ensured. Each interview lasted between 30 and 40 min. All interviews were audio taped with permission from the women, and transcribed verbatim for analysis.

Data analysis was concurrent with data collection. The accuracy of the transcribed transcripts was checked by repeated, simultaneous reading of transcripts, and listening to the audiotape. After all audio-recordings had been transcribed, thematic content analysis was conducted [14]. Thematic content analysis involves the process of labelling qualitative information to identify and interpret patterns in raw text. In thematic analysis, the researcher familiarizes with the transcript by reading carefully in order to identify the themes. Therefore, when the researchers were familiar with the transcripts, the theme was derived based on the constructs of the psychological component of the bio-psychosocial model using a carefully developed thematic code frame. All phrases or sentences that fitted a particular code were labelled as such.

The researchers reviewed the theme and emerged sub-themes, and the relationships among categories were used to describe the psychological experiences of infertility among these women.

## Results

The findings showed that women with fertility problems encountered some emotional stressors. Psychological experience was identified as the main theme; six additional subthemes identified were loneliness, anxiety, depression, lack of concentration, worrying, and reduced sexual satisfaction. The findings have been presented with verbatim quotations from the women.

### Demographic characteristics

Table 1 shows the details of the demographic characteristics of the women. The ages of the women ranged from 27 to 42 years. However, the majority (8, 57%) were between 27 and 32 years. The majority (12, 86%) had lived with infertility for two to five years.

**Table 1:** Demographic Characteristics of Participants

Characteristics	Frequency	Percent (%)
Age (years)		
27–32	8	57
33–37	4	29
38–42	2	14
Duration of infertility (years)		
2–5	12	86
6–9	1	7
10–13	1	7
Marital status Married	14	100
Number of children		
No children	12	86
At least one child	2	14
Education		
No formal education	1	7
Primary	7	50
Secondary	1	7
Tertiary	5	36
Employment		
Government	6	36
Private	9	64

All the women (14, 100%) were married. A total of 12 (86%) of the women had no child at all whilst the rest (2, 14%) had one child each and were looking forward to having more. With regard to occupation, the majority (9, 64%) were self-employed or engaged in private businesses whilst the remaining 36% (*n* = 5) were government employees. The women had varied levels of formal education. Half (7,50%) of the women had primary level of education and 5(36%) had tertiary level of education.

### Psychological experiences

Psychological experiences emerged as the main theme with loneliness, anxiety, depression, lack of concentration, worrying and reduced sexual satisfaction as subthemes. Below are the meanings of these psychological experiences as transcribed verbatim from the participants’ responses.

### Loneliness

This was experienced by the women in diverse forms. Most of the women expressed that they felt very lonely when they came home from work and realized their homes were without a cry or a sound of a baby. Others shared that sometimes they wished after close of work, they could involve themselves in extra chores like changing of diapers or cuddling a baby, as lamented below:

*I am always lonely and sometimes imagine how life would have been with a child. I sometimes think that if I had a child, I would have been cuddling or playing with it or changing its diapers. I have always bargained with God for a pregnancy even if it ends up in a miscarriage. At least with that, I can say that I got pregnant and miscarried. However, this has never been the case during my three (3) years of marriage. The desire for a child is immense. I don’t know why I am going through all this.*

Two women also shared their stories in different ways. They shared these:

*I do experience loneliness sometimes and these are on days when my husband has travelled and I am all alone in the house. I wished I had a child by me at least to be talking to, playing with or even sending on errands but I look around and not even a sign of a child’s…cry,* she sighs.

*I experience much loneliness especially on days when my husband isn’t around. I could cry my heart out until I got consolation from him, I did not stop crying*.

One other woman bitterly shared that, what triggered her loneliness more was how her in-laws maltreated her:

*I experience a lot of loneliness. My sister-in-law uses my inability to have children as grounds to hurt my feelings whenever we have a misunderstanding in order to silence me. Other times, she will carry one of her children and say to me, “can’t you see that I have brought children to this home? Where are yours?*

### Anxiety

Anxiety stemmed from the fact that most women felt they were aging but showed no sign of pregnancy. Hence, they were scared of becoming old without carrying their own children. These fears were also coupled with the fact that their friends who got married after them have their own children. Two of them had this to share:

*Sometimes, I get very anxious when I cast my mind back and see that all who got married after me are having children. It appears I am the only one left behind*.

*I am aging, I am aging… Very soon I will get to the pre-menopausal stage. What will I do when it happens? I cannot even brag of a child.*

Fear of losing a husband was also one of the reasons why the women experienced anxiety. One (1) woman shared that, because the custom of her husband and herself promotes polygamy, she feared she may lose her husband to other women who can give him a child. This is what she had to say:

*Being a Muslim, you know that in our tradition, a man can marry more than one (1) wife and because of this, sometimes I feel if I do not try my very best to get myself pregnant, my husband will one day go in for another woman to give him children although he has not expressed that to me yet*.

### Depression

Feelings of depression among some of the women were ascribed to the fact that they have been married for some years and there is still no sign of pregnancy. Other women expressed that the humiliation from colleagues and friends at both the work place and home made them feel very depressed. A woman expressed her challenge as this:

*I am always depressed. After three (3) whole years of marriage, the society expects that I either get pregnant or at least carry my own baby and since I have failed to achieve these, anybody at all speaks to me anyhow without regard.*

Another woman bitterly expressed her ordeal as this:

*I am always depressed and worried. Madam, my fellow women make me feel very bad and sorrowful. Sometimes in my own shop people come over and accuse me of having chewed all the babies in my womb. Some even go to the extent of calling my eleven (11) year old boy “jimi…jimi”,* meaning a fool*. Since people feel because he is my only child, he acts like an over pampered imbecile.*

Another woman also attributed her state of depression to the fact that she could not control her tears any time she heard her friends and colleagues speak about their children in her presence. She had this to share:

*When I come back from work and realize no one is around, I cry throughout, especially when at work I hear colleagues and friends talking about their children. In fact my situation has made me very depressed.*

### Lack of concentration

Lack of concentration experienced by the women with fertility problems had to do with the fact that they sometimes got carried away by their challenges. Some women reported not being able to concentrate in most cases. This is what a woman had to say:

*I sometimes lose concentration to the extent that, I even forget to eat at times*.

Another participant also expressed her lack of concentration as follows:

*Sometimes, I think incessantly to the extent that I lose concentration.*

Another woman described her feelings of lack of concentration this way:

*Sometimes when I sit down, I unconsciously talk to myself as though I were mad.*

### Worrying

Worrying was another major challenge these women had to battle with. These were quiet, intense and based on varied reasons. Some were worried because of the fact that their colleagues and age mates got married and had children while they did not have the latter. Others also attributed their worries to the fact that, they always felt humiliated among people. Others attributed their worry to their husbands mounting pressure on them as described below:

*In fact I am very worried because I do not know what I have done to Allah to incur such punishment*.

*I am being treated harshly for a problem that I did not bring upon myself. I am treated as though I were a nobody and that hurts me a lot*.

In the words of another woman, sometimes the pressure meted by her husband made her feel worried and stressed. She described how she felt:

*My husband always says that, “Maame” we have kept long, when at all are we also going to give birth and have our own children? In fact I feel very disturbed and worried and therefore wish the children of other people were my own blood*.

### Reduced sexual satisfaction

Most of the women stated that they experienced reduced sexual satisfaction because they believed that if all these years of love making could not make them pregnant, they did not see the need to engage in subsequent love making. Other women expressed their decreased sexual satisfaction as quoted below:

*I do not really enjoy sex with my husband and this is because in my heart and mind, I feel that if after all these years of regular intercourse, I have not been able to get pregnant then there is no need to enjoy sex since it does not result in childbirth. Sometimes I am not even sexually aroused since I do not see the need for sex.*

Another woman also expressed this:

*Sometimes when my husband wants to make love to me, I feel he is bothering me. I have this notion that, if all these years of love making, nothing really came out of it, then there is no reason to keep bothering each other with sex. Due to this and other thoughts that run through my mind, I always experience reduced sexual satisfaction*.

In summary, the psychological experiences of women with infertility are diverse based on their beliefs and cultural orientations. These experiences are all negative suggesting that these women may have poor mental health.

## Discussion

The demographic characteristics of the women suggest that the majority of them were within the fertile age. However, anxiety and depression were the notable psychological problems reported. A possible explanation to this finding could be that childbearing is a socio-cultural expectation of the Ghanaian society after marriage. Furthermore, the experience of infertility for two to five years is enough duration for a typical Ghanaian woman to exhibit psychological problems.

Infertility is a major public health problem that can result in psychological consequences for couples, especially women. These women reported experiencing loneliness, anxiety, depression, lack of concentration, worrying, and decreased sexual satisfaction.

Loneliness is referred to as a state of complex and unpleasant emotional response to isolation or lack of companionship. The feeling of loneliness among these women was due to associated interpersonal problems. For instance the mere fact that they closed from work, got home and there was no sign of a child, made them feel traumatized. Loneliness was also attributed to the fact that there was a change of attitude of their husbands. This negative attitude of their husbands put them in a state of isolation, which is consistent with experiences of women in Turkey [15].

Findings from the current study again revealed that women with fertility problems suffered anxiety as reported among women in Karachi-Pakistan [11]. In the current study, the reason assigned by most women for being anxious varied from one woman to the other, including the duration of marriage without any sign of pregnancy. Furthermore, advancing age was another reason why these women often got anxious. Additionally, fear of losing their husbands to other women was another major concern in the lives of these women. However, anxiety among women with infertility is inconsistently reported in the literature. For instance, a study conducted in Ghana revealed that although women with fertility problems encountered higher levels of infertility-related stress, they experienced lower levels of anxiety [6]. As a result, further research is needed to investigate the factors associated with infertility-related anxiety in Ghana.

Depression is a feeling of sadness and rejection. It is indeed considered as one of the most common psychological disorders affecting couples with fertility problems, more especially women [13]. Another aspect of depression reported by the women was the feeling of humiliation, and insults, which led to extreme sadness and sorrow. These are consistent with previous findings in the literature [12]. Reduced sexual satisfaction is another psychological problem reported by the women, which ties in with the literature [8, 16, 17]. Thus, women with fertility problems are faced with several psychological problems that have devastating effects on their mental health as well as their overall well-being.

This study has the strength of reporting a number of psychological problems related to infertility. However, these findings are limited since data were collected from only one tertiary hospital in the Greater Accra region of Ghana, and only 14 women were interviewed. Therefore, transferability of the findings may only be possible when the population and the context are similar.

## Conclusion

Infertility has a negative influence on the psychological well-being of women. Infertile women experienced psychological problems such as loneliness, anxiety, depression, lack of concentration, worrying, and reduced sexual satisfaction. Consequently, there is the need for psychological counseling for these women in order to mitigate their psychological challenges as recommended by the bio-psychosocial model.
